# Vascular, inflammatory and metabolic risk factors in relation to dementia in Parkinson’s disease patients with type 2 diabetes mellitus

**DOI:** 10.18632/aging.103776

**Published:** 2020-08-15

**Authors:** Ting Wang, Feilan Yuan, Zhenze Chen, Shuzhen Zhu, Zihan Chang, Wanlin Yang, Bin Deng, Rongfang Que, Peihua Cao, Yinxia Chao, Lingling Chan, Ying Pan, Yanping Wang, Linting Xu, Qiurong Lyu, Piu Chan, Midori A. Yenari, Eng-King Tan, Qing Wang

**Affiliations:** 1 Department of Neurology, Zhujiang Hospital of Southern Medical University, Guangzhou, Guangdong, P.R. China; 2 Clinical Research Center, ZhuJiang Hospital of Southern Medical University, Guangzhou, Guangdong, P.R. China; 3 Department of Neurology, National Neuroscience Institute, Singapore General Hospital, Duke-NUS Medical School, Singapore; 4 Department of Neurology, The Second Affiliated Hospital of Guangzhou Medical University, Guangzhou, Guangdong, China; 5 Department of Neurology, Puning People’s Hospital, Puning, Guangdong, China; 6 Department of Neurology, Guiping People’s Hospital, Guangxi, China; 7 Department of Neurology, Xuanwu Hospital of Capital Medical University, Beijing, China; 8 Department of Neurology, University of California, San Francisco and the San Francisco Veterans Affairs Medical Center, San Francisco, CA 94121, USA

**Keywords:** vascular inflammation, risk factor, dementia, Parkinson's disease, type 2 diabetes mellitus

## Abstract

There are limited data on vascular, inflammatory, metabolic risk factors of dementia in Parkinson’s disease (PD) with type 2 diabetes mellitus (DM) (PD-DM). In a study of 928 subjects comprising of 215 PD with DM (including 31 PD-DM with dementia, PD-DMD), 341 PD without DM (including 31 PD with dementia, PDD) and 372 DM without PD (including 35 DM with dementia, DMD) patients, we investigated if vascular, inflammatory, metabolic, and magnetic resonance imaging (MRI) markers were associated with dementia in PD-DM. Lower fasting blood glucose (FBG<5mmol/L, OR=4.380; 95%CI: 1.748-10.975; p=0.002), higher homocysteine (HCY>15*μ*mol/L, OR=3.131; 95%CI: 1.243-7.888; p=0.015) and hyperlipidemia (OR=3.075; 95%CI: 1.142-8.277; p=0.026), increased age (OR=1.043; 95%CI: 1.003-1.084; p=0.034) were the most significant risk factors in PDD patients. Lower low-density lipoprotein cholesterol (LDL-C<2mmol/L, OR=4.499; 95%CI: 1.568-12.909; p=0.005) and higher fibrinogen (>4g/L, OR=4.066; 95%CI: 1.467-11.274; p=0.007) were the most significant risk factors in PD-DMD patients. The area under the curve (AUC) for fibrinogen and LDL-C was 0.717 (P=0.001), with a sensitivity of 80.0% for the prediction of PD-DMD.

In summary, we identified several factors including LDL-C and fibrinogen as significant risk factors for PD-DMD and these may have prognostic and treatment implications.

## INTRODUCTION

Patients with type 2 diabetes mellitus (DM) may have a higher risk of developing Parkinson’s disease (PD) [1]. Patients with comorbid PD and DM (PD-DM) are commonly encountered in clinical practice. Cognitive dysfunction in this group of patients may contribute to the underlying neurodegenerative process and impact on quality of life [2, 3]. Vascular, inflammatory and metabolic derangements can potentially modulate the underlying pathophysiologic processes in PD [4–7]. In fact, glucose metabolism abnormalities (including insulin resistance) have been observed in 50-80% of patients with PD [8–10]. PD-DM patients have been previously associated with cognitive decline or dementia [11, 12]. The mechanism of dementia in PD-DM (PD-DMD) patients has yet to be elucidated.

Several lines of evidence suggested that altered blood glucose could lead to cognitive impairment in old age [13], and patients with PD-DM are more prone to develop cognitive impairment than patients with PD but without DM [14]. In addition, various vascular, inflammatory [15, 16] and metabolic markers, including cystatin C (Cys C), homocysteine (HCY), low-density lipoprotein (LDL-C), neutrophils, lymphocytes, white matter lesions (WMLs) and subcortical arteriosclerotic encephalopathy (SAE), have been shown to be associated with dementia in PD and DM [12, 17, 18]. It is possible that vascular, inflammatory and metabolic risk factors in PD can modulate underlying neurodegeneration particularly in the presence of diabetes and dementia [19–21]. However, among these risk factors, which risk factors could be mostly associated with dementia and if these risk factors could be potential prognostic clinical variables to facilitate the early prediction of PD-DMD patients have not been reported and need to be further explored.

To address these gaps in knowledge, we conducted a large cohort study to examine the relationship between vascular, inflammatory, metabolic risk factors and dementia in PD-DM patients. We also identified the potential and most significant risk factors for PD-DMD, with the aim of validating the potential prognostic clinical variables to facilitate the early prediction of PD-DMD patients.

## RESULTS

### Baseline demographics in PD, DM and PD-DM patients

**Figure 1:**
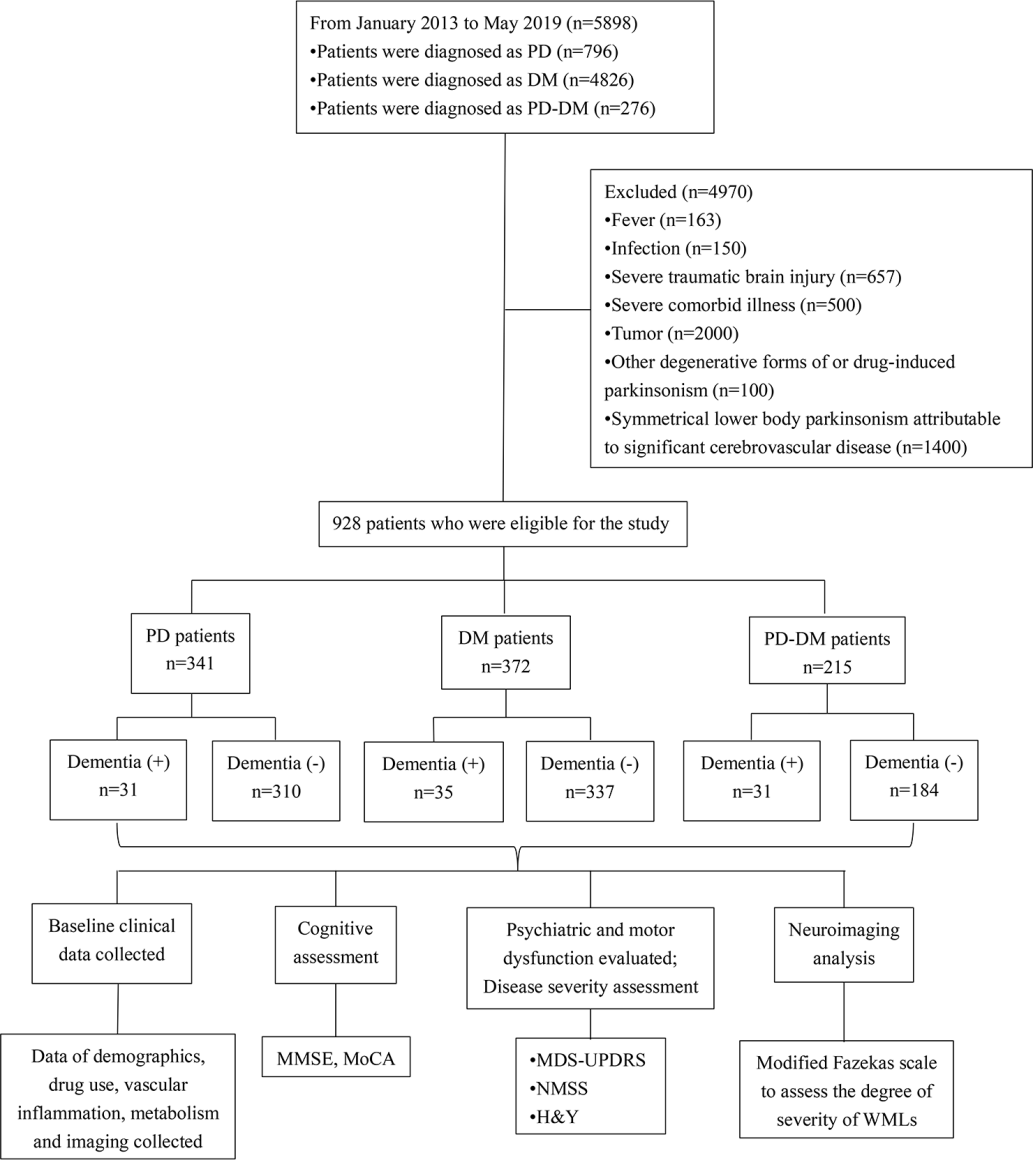
**Flow diagram of patients with dementia diagnosed with PD, DM, or PD-DM and the clinical investigations conducted.** PD - patients with Parkinson’s disease without type 2 diabetes mellitus; DM - type 2 diabetes mellitus without Parkinson’s disease; PD-DM - patients with Parkinson’s disease and type 2 diabetes mellitus; MDS-UPDRS - Movement Disorder Society–Unified Parkinson’s Disease Rating Scale; H&Y - the modified Hoehn and Yahr staging scale; NMSS - nonmotor symptoms scale for Parkinson’s Disease; MMSE - Mini Mental State Examination; MoCA - Montreal Cognitive Assessment; WMLs - White matter lesions.

**Figure 2:**
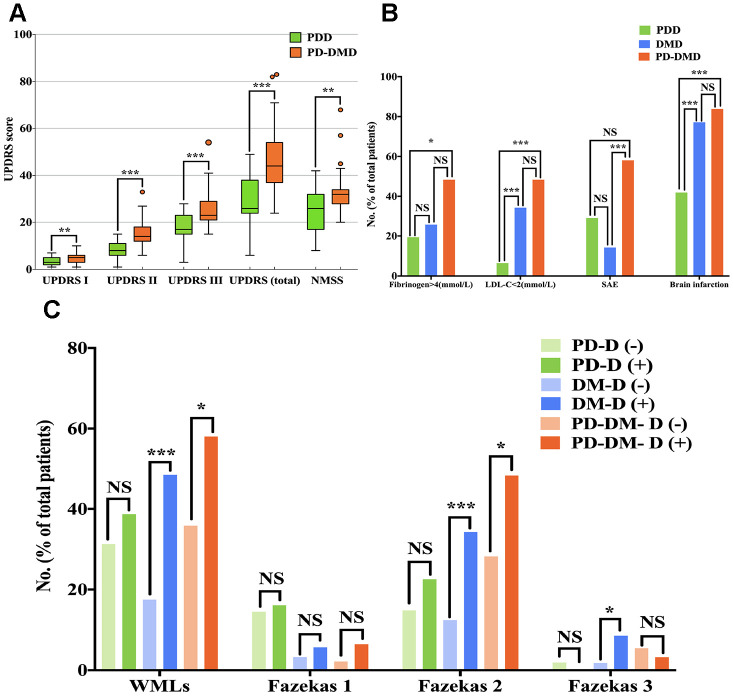
Comparison of UPDRS score (**A**) and the percentage of patients with fibrinogen>4 (mmol/L), LDL<2 (mmol/L), SAE, brain infarctions (**B**) and WMLs with Fazekas scale scores (**C**) among PDD, DMD and PD-DMD patients. PD - patients with Parkinson’s disease without type 2 diabetes mellitus; DM - type 2 diabetes mellitus without Parkinson’s disease; PD-DM - patients with Parkinson’s disease and type 2 diabetes mellitus; PDD - patients with Parkinson’s disease with dementia; DMD - type 2 diabetes mellitus with dementia; PD-DMD - patients with Parkinson’s disease and type 2 diabetes mellitus and dementia; D(-) - without dementia; D(+) - with dementia; UPDRS - Unified Parkinson’s Disease Rating Scale; NMSS - nonmotor symptoms scale for Parkinson’s Disease; LDL-C - low-density lipoprotein cholesterol; SAE - subcortical arteriosclerotic encephalopathy; WMLs - white matter lesions; NS - not significant. *p<0.05, **p<0.01, ***p<0.001.

The baseline demographics in PD, DM and PD-DM patients are summarized in Table 1 and Figure 1. A total of 928 patients comprising of 341 PD (31 PD with dementia, PDD), 372 DM (35 DM with dementia, DMD) and 215 PD-DM (31 PD with dementia, PD-DMD) patients were recruited (Fig 1). No significant difference in the proportion of dementia cases among PD, DM and PD-DM patients was noted (Table 1). The numbers of patients with PD-DMD and DMD over 70 years were greater than the number of patients without dementia (Table 1). Among all these patients with dementia, the numbers of PD-DMD patients over 70 years were significantly more than those of PDD and DMD patients (Supplementary Table 2). PD-DMD and PDD patients exhibited higher UPDRS and NMSS scores than patients without dementia; PD-DMD patients exhibited higher H&Y stages and more use of atorvastatin than patients without dementia (Table 1). Among all of these patients with dementia, PD-DMD patients exhibited higher MDS-UPDRS and NMSS scores than patients with PDD (Figure 2, Supplementary Table 2). PD-DMD and DMD patients exhibited more anxiety and depression than patients without dementia. DMD patients were more often male and exhibited more acarbose use but less insulin use than DM patients without dementia (p<0.05). However, there was no significant difference in the use of metformin between DM and PD-DM patients or in the use of L-Dopa and PD duration between PD and PD-DM patients with dementia and those without dementia (Table 1). We found no significant difference in MMSE, MoCA, or H&Y scores, the proportion of males, the presence of anxiety or depression, or the use of insulin and acarbose among the PDD, DMD and PD-DMD patients (Supplementary Table 2).

**Table 1:** Baseline demographics in PD, DM and PD-DM patients.

**Clinical variables**	**PD(n=341)**				**DM(n=372)**				**PD-DM(n=215)**		
	**D (-) (n=310)**	**D (+) (n=31)**	p*		**D (-) (n=337)**	**D (+) (n=35)**	p*		**D (-) (n=184)**	**D (+) (n=31)**	p*
**Age, years**	65.0(56.0,73.0)	69.0(59.0,79.0)	0.082		**61.0(52.0,69.0)**	**68.0(57.0,74.0)**	**0.013**		**73.0(66.0,80.0)**	**79.0(72.0,84.0)**	**0.004**
<55, n (%)	60(19.35)	3(9.68)	0.186		109(32.34)	7(20.00)	0.133		9(4.89)	0	0.364
55–70, n (%)	156(50.32)	16(51.61)	0.891		148(43.92)	14(40.00)	0.198		**64(34.78)**	**5(16.13)**	**0.040**
>70, n (%)	94(30.32)	12 (38.71)	0.336		**80(23.74)**	**14(40.00)**	**0.035**		**111(60.33)**	**26(83.87)**	**0.012**
**Male, n (%)**	175(56.45)	18(58.06)	0.863		**192(56.97)**	**27(77.14)**	**0.021**		88(47.83)	16(51.61)	0.696
**PD duration, month**	48 (16.5,84)	60(24,84)	0.260		-	-	-		36(12,96)	36(12,72)	0.363
**Use of drugs, n (%)**											
Atorvastatin	67(21.61)	6(19.35)	0.770		178(52.82)	14(40.00)	0.149		**83(45.11)**	**20(64.52)**	**0.045**
Metformin	-	-	-		181(53.71)	14(40.00)	0.122		74(40.22)	9(29.03)	0.237
Insulin	-	-	-		**171(50.74)**	**8(22.86)**	**0.002**		69(37.50)	14(45.16)	0.418
Acarbose	-	-	-		**94(27.89)**	**17(48.57)**	**0.011**		78(42.39)	18(58.06)	0.104
L-Dopa	252(81.29)	28(90.32)	0.211		-	-	-		143(77.72)	26(83.87)	0.440
**MDS-UPDRS (I)**	**1.0(0.0,2.0)**	**3.0(2.0,5.0)**	**<0.001**		**-**	**-**	**-**		**2.0(1.0,4.0)**	**5.0(3.0,6.0)**	**<0.001**
**MDS-UPDRS (II)**	**7.0(5.0,8.0)**	**8.0(6.0,11.0)**	**0.017**		**-**	**-**	**-**		**9.0(7.0,14.0)**	**14.0(12.0,18.0)**	**<0.001**
**MDS-UPDRS (III)**	17.0(15.0,19.0)	17.0(15.0,23.0)	0.610		-	-	-		**21.0(17.0,27.0)**	**23.0(21.0,29.0)**	**0.023**
**MDS-UPDRS (Total)**	**25.0(21.0,29.0)**	**26.0(24.0,38.0)**	**0.016**		**-**	**-**	**-**		**34.0(28.0,44.8)**	**44.0(37.0,54.0)**	**<0.001**
**H&Y**	3.0(2.0,3.0)	3.0(2.0,4.0)	0.236		-	-	-		**3.0(2.5,3.0)**	**3.0(3.0,4.0)**	**0.008**
**NMSS**	**18.0(13.8,24.0)**	**26.0(17.0,32.0)**	**<0.001**		**-**	**-**	**-**		**24.0(18.0,32.0)**	**32.0(28.0,34.0)**	**<0.001**
**MMSE**	**26.0(24.0,28.0)**	**15.0(13.0,17.0)**	**<0.001**		**27.0(26.0,29.0)**	**15.0(9.0,19.0)**	**<0.001**		**26.0(24.0,27.0)**	**13.0(11.0,17.0)**	**<0.001**
**MoCA**	**23.0(20.0,25.0)**	**12.0(9.0,14.0)**	**<0.001**		**26.0(24.0,27.0)**	**12.0(7.0,15.0)**	**<0.001**		**22.0(20.0,24.0)**	**9.0(8.0,13.0)**	**<0.001**
**Anxiety or depression, n(%)**	131(42.26)	17(54.84)	0.178		**52(15.43)**	**13(37.14)**	**0.001**		**76(41.30)**	**19(61.29)**	**0.038**

All continuous variables are presented as median (interquartile range) and categorical variables are presented as count (proportion). PD- patients with Parkinson disease without type 2 diabetes mellitus; DM- type 2 diabetes mellitus without Parkinson disease; PD-DM- patients with Parkinson disease and type 2 diabetes mellitus; D(-)- without dementia; D(+)- with dementia; L-Dopa- Levodopa and Benserazide; MDS-UPDRS- Movement Disorder Society–Unified Parkinson’s Disease Rating Scale; H&Y- the modified Hoehn and Yahr staging scale; NMSS- Non-Motor Symptoms Scale for Parkinson’s Disease; MMSE- mini mental state examination; MoCA- Montreal Cognitive Assessment; *The statistically significant differences between patients with dementia and patients without dementia in PD, DM, PD-DM groups were assessed by χ2-test or Mann-Whitney U tests; P-values<0.05 were considered statistically significant.

### Vascular and inflammatory risk factors for dementia in PD, DM and PD-DM patients

The potential vascular and inflammatory risk factors for dementia in PD, DM and PD-DM patients are summarized in Table 2A. We found that PD-DMD and DMD patients exhibited higher plasma levels of fibrinogen (>4g/L) and neutrophils, and more brain infarctions, SAE and WMLs (Fazekas 2), but lower levels of LDL-C (<2mmol/L) and lymphocytes, than PD-DM and DM patients without dementia (p<0.05), whereas no significant differences were noted in PD patients. Among all patients with dementia, PD-DMD and DMD patients exhibited more brain infarctions but lower levels of LDL-C (<2mmol/L) than PDD patients; PD-DMD patients exhibited higher levels of neutrophils and fibrinogen (>4g/L) but lower levels of lymphocytes than PDD patients. PD-DMD patients exhibited more SAE than DMD patients; DMD patients exhibited higher levels of hs-CRP (>3mg/L) than PDD patients (p<0.05) (Supplementary Table 2, Figure 2). However, there was no significant difference in blood pressure, BMI, WBC, or the incidence of smoking history, drinking history, or hyperlipidemia between patients (PD, DM and PD-DM patients) with dementia and those without dementia. There was no significant difference in the level of D-dimer or the proportion of WMLs among PDD, DMD and PD-DMD patients.

**Table 2A:** Vascular and inflammatory risk factors for dementia in PD, DM and PD-DM patients.

**Vascular and inflammatory risk factors**	**PD(n=341)**				**DM(n=372)**				**PD-DM(n=215)**		
	**D (-) (n=310)**	**D (+) (n=31)**	p*		**D (-) (n=337)**	**D (+) (n=35)**	p*		**D (-) (n=184)**	**D (+) (n=31)**	p*
**SBP (mmHg)**	125.0(120.0,135.0)	120.0(118.0,135.0)	0.104		136.0(124.0,151.0)	146.0(128.0,153.0)	0.104		137.0(125.0,151.0)	132.0(120.0,152.0)	0.333
**DBP (mmHg)**	80.0(75.0,82.0)	80.0(78.0,81.0)	0.974		81.0(76.5,90.0)	85.0(80.0,95.0)	0.196		77.0(70.0,84.0)	78.0(69.0,87.0)	0.821
**Smoking history, n (%)**	14(4.52)	2(6.45)	0.647		48(14.24)	7(20.00)	0.361		11(5.98)	3(9.68)	0.432
**Drinking history, n (%)**	10(3.23)	0	0.608		30(8.90)	5(14.29)	0.355		6(3.26)	1(3.23)	1.000
**BMI**	22.5(21.0,25.0)	23.0(22.0,25.0)	0.064		24.0(22.0,26.0)	24.0(22.0,26.0)	0.823		23(21,26)	24(21,25)	0.710
**LDL-C (mmol/L)**	2.9(2.3,3.5)	2.5(2.3,3.2)	0.270		**3.1(2.4,3.6)**	**2.7(1.9,3.1)**	**0.010**		**2.8(2.1,3.5)**	**1.9(1.6,3.0)**	**0.009**
LDL-C<2.00 (mmol/L), n (%)	31(10.00)	2(6.45)	0.753		**67(19.88)**	**12(34.29)**	**0.047**		**36(19.57)**	**15(48.39)**	**<0.001**
**D-Dimer (mg/L)**	0.4(0.3,0.6)	0.6(0.3,1.1)	0.083		0.4(0.3,0.5)	0.4(0.3,0.9)	0.311		0.6(0.4,1.4)	0.6(0.4,1.4)	0.622
D-Dimer>0.50 (mg/L), n (%)	57(18.39)	10(32.26)	0.064		**25(7.42)**	**8(22.86)**	**0.007**		50(27.17)	10(32.26)	0.559
**Fibrinogen (g/L)**	3.1(2.7,3.5)	3.5(2.8,4.0)	0.052		3.5(3.0,4.1)	3.3(3.0,4.6)	0.863		**3.5(3.0,4.4)**	**4.1(3.5,5.1)**	**0.015**
Fibrinogen>4.00 (g/L), n (%)	27(8.71)	6(19.35)	0.101		**42(12.46)**	**9(25.71)**	**0.039**		**45(24.46)**	**15(48.39)**	**0.006**
**WBC (g/L)**	6.3(5.3,7.4)	6.1(5.0,7.2)	0.533		6.9(6.0,8.3)	7.3(6.0,9.5)	0.152		7.2(6.1,8.8)	7.4(6.1,9.9)	0.387
**Lymphocyte (%)**	30.4(25.8,35.9)	30.7(24.2,34.8)	0.740		**30.9(25.2,37.5)**	**25.3(18.6,32.1)**	**0.002**		**27.3(19.0,32.6)**	**20.5(12.2,28.0)**	**0.008**
**Neutrophil (%)**	58.3(52.3,64.3)	58.6(52.5,67.5)	0.860		**58.7(52.5,65.5)**	**64.0(58.3,70.2)**	**0.001**		**63.2(57.2,71.8)**	**71.5(61.6,82.8)**	**0.007**
**hs-CRP (mg/L)**	1.1(0.6,4.1)	0.6(0.6,2.0)	0.094		**1.8(0.6,5.2)**	**3.6(1.5,7.1)**	**0.019**		3.6(0.7,10.6)	4.5(0.7,47.1)	0.861
hs-CRP>3.00 (mg/L), n (%)	34(10.97)	2(6.45)	0.757		**52(15.43)**	**11(31.43)**	**0.016**		43(23.37)	8(25.81)	0.768
**Hyperlipidemia, n(%)**	33(10.65)	7(22.58)	0.072		61(18.10)	3(8.57)	0.155		24(13.04)	2(6.45)	0.385
**Brain infarction, n(%)**	90(29.03)	13(41.94)	0.140		**128(37.98)**	**27(77.14)**	**<0.001**		**86(46.73)**	**26(83.87)**	**<0.001**
**SAE, n(%)**	70(22.58)	9(29.03)	0.417		**13(3.86)**	**5(14.29)**	**0.019**		**48(26.09)**	**18(58.06)**	**<0.001**
**WMLs, n (%)**	97(31.29)	12(38.71)	0.398		**59(17.51)**	**17(48.57)**	**<0.001**		**66(35.87)**	**18(58.06)**	**0.019**
Fazekas 1	45(14.52)	5(16.13)	0.791		11(3.26)	2(5.71)	0.350		4(2.17)	2(6.45)	0.208
Fazekas 2	46(14.84)	7(22.58)	0.295		**42(12.46)**	**12(34.29)**	**<0.001**		**52(28.26)**	**15(48.39)**	**0.025**
Fazekas 3	6(1.94)	0	1.000		**6(1.78)**	**3(8.57)**	**0.043**		10(5.43)	1(3.23)	1.000

All continuous variables are presented as median (interquartile range) and categorical variables are presented as count (proportion). PD- patients with Parkinson disease without type 2 diabetes mellitus; DM- type 2 diabetes mellitus without Parkinson disease; PD-DM- patients with Parkinson disease and type 2 diabetes mellitus; D(-)- without dementia; D(+)- with dementia; SBP- systolic blood pressure; DBP- diastolic blood pressure; BMI- body mass index; LDL-C- low density lipoprotein cholesterol; WBC- White blood cell count; hs- CRP- hypersensitive C-reactive protein; SAE- subcortical arteriosclerotic encephalopathy; WMLs- White matter lesions. *The statistically significant differences between patients with dementia and patients without dementia in PD, DM, PD-DM groups were assessed by χ2-test or Mann-Whitney U tests; P-values<0.05 were considered statistically significant.

### Metabolic risk factors for dementia in PD, DM and PD-DM patients

The metabolic risk factors for dementia in PD, DM and PD-DM patients are summarized in Table 2B. First, we found a higher plasma level of Cys C (>0.95mg/L, p<0.05) in PDD and DMD patients than in PD and DM patients without dementia, whereas no significant differences were observed between PD-DMD and PD-DM patients without dementia. Second, higher levels of MCV (>90fL) and HCY (>15*μ*mol/L), more frequent hyperhomocysteinemia, and lower levels of FBG (<5mmol/L) were observed in PDD patients than in PD patients without dementia. Third, higher plasma levels of AST (>40IU/L) and albumin (<35g/L) but lower levels of calcium (<2.1mmol/L) and potassium were observed in DMD patients than in DM patients without dementia (Table 2B). Higher levels of MCV (>90fL) but lower levels of FBG (<5mmol/L) were noticed in PDD patients than in patients with PD-DMD or DMD (Supplementary Table 2). However, no significant differences in ALT or HbA1c levels were noted between patients (PD, DM and PD-DM patients) with dementia and without dementia. There was no significant difference in AST, albumin, Cys C, HCY, calcium or potassium levels among PDD, DMD and PD-DMD patients.

**Table 2B:** Metabolic risk factors for dementia in PD, DM and PD-DM patients.

**Metabolic risk factors**	**PD(n=341)**				**DM(n=372)**				**PD-DM(n=215)**		
	**D (-) (n=310)**	**D (+) (n=31)**	p*		**D (-) (n=337)**	**D (+) (n=35)**	p*		**D (-) (n=184)**	**D (+) (n=31)**	p*
**AST>40(IU/L), n (%)**	7(2.26)	0	1.000		**9(2.67)**	**5(14.29)**	**0.006**		14(7.61)	3(9.68)	0.718
**ALT(IU/L)**	14.0(10.0,21.0)	15.0(9.0,22.0)	0.782		18.0(13.0,27.0)	22.0(15.0,28.0)	0.138		15.0(10.0,22.0)	14.0(11.0,30.0)	0.350
**Albumin (g/L)**	40.5(38.0,43.0)	40.3(38.0,41.8)	0.575		**41.4(39.0,43.9)**	**39.3(36.2,42.9)**	**0.020**		39.8(37.0,42.0)	36.9(36.0,42.1)	0.166
Albumin<35.00 (g/L), n (%)	14(4.52)	2(6.45)	0.647		**8(2.37)**	**4(11.43)**	**0.019**		27(14.67)	7(22.58)	0.288
**Calcium (mmol/L)**	2.3(2.2,2.3)	2.3(2.2,2.4)	0.529		**2.3(2.2,2.4)**	**2.2(2.2,2.3)**	**0.004**		2.3(2.2,2.4)	2.2(2.1,2.3)	0.134
Calcium<2.10 (mmol/L), n (%)	28(9.03)	5(16.13)	0.203		**12(3.56)**	**6(17.14)**	**0.004**		24(13.04)	8(25.81)	0.097
**Potassium (mmol/L)**	3.9(3.7,4.1)	3.8(3.6,4.1)	0.159		**4.0(3.7,4.2)**	**3.9(3.6,4.0)**	**0.041**		3.9(3.6,4.3)	3.8(3.4,4.3)	0.289
**MCV (fL)**	**91.0(88.2,93.9)**	**92.7(91.1,97.4)**	**0.003**		88.1(85.1,90.6)	87.2(83.5,93.3)	0.866		89.9(86.9,93.2)	89.9(88.2,93.2)	0.700
MCV>90.00 (fL), n (%)	**187(60.32)**	**25(80.65)**	**0.026**		103(30.56)	13(37.14)	0.424		88(47.83)	14(45.16)	0.783
**HCY (*μ*mol/L)**	13.4(10.7,17.0)	15.8(12.0,17.8)	0.177		11.2(9.2,14.5)	11.3(8.5,15.3)	0.973		12.6(9.5,15.8)	12.3(8.7,18.0)	0.714
HCY>15.00 (*μ*mol/L), n (%)	**41(13.23)**	**9(29.03)**	**0.029**		36(10.68)	5(14.29)	0.568		24(13.04)	5(16.13)	0.580
**Hyperhomocysteinemia, n (%)**	**11(3.55)**	**4(12.90)**	**0.038**		5(1.48)	0	1.000		2(1.09)	1(3.23)	0.375
**Cys C (mg/L)**	**1.0(0.9,1.1)**	**1.1(1.0,1.2)**	**0.031**		0.9(0.8,1.0)	1.0(0.9,1.1)	0.066		1.1(0.9,1.3)	1.0(0.9,1.5)	0.834
Cys C>0.95 (mg/L), n (%)	**95(30.65)**	**15(48.39)**	**0.044**		**24(7.12)**	**8(22.86)**	**0.006**		48(26.09)	7(22.58)	0.679
**FBG (mmol/L)**	**4.9(4.6,5.4)**	**4.7(4.3,5.1)**	**0.004**		8.2(6.2,12.0)	7.6(5.8,11.6)	0.275		7.4(5.9,9.1)	7.1(6.1,10.0)	0.756
FBG<5.00 (mmol/L), n (%)	**151(48.71)**	**23(74.19)**	**0.007**		34(10.09)	5(14.29)	0.393		21(11.41)	1(3.23)	0.213
**Hypoglycaemic episodes** (FBG≤3.9mmol/L), n (%)	6(1.94)	1(3.23)	0.490		**6(1.78)**	**3(8.57)**	**0.043**		3(1.63)	0	1.000
**HbA1c (%)**	5.8(5.5,6.1)	5.7(5.2,5.9)	0.197		7.8(6.6,9.7)	7.3(6.3,8.9)	0.369		8.0(7.1,9.0)	6.4(6.0,7.4)	0.101

All continuous variables are presented as median (interquartile range) and categorical variables are presented as count (proportion). PD- patients with Parkinson disease without type 2 diabetes mellitus; DM- type 2 diabetes mellitus without Parkinson disease; PD-DM- patients with Parkinson disease and type 2 diabetes mellitus; D(-)- without dementia; D(+)- with dementia; AST- Aspartate transaminase; ALT- Alanine transaminase; MCV- mean corpuscular volume; HCY- homocysteine; Cystatin C- Cys C; FBG- Fasting blood glucose; HbA1c- glycated hemoglobin; *The statistically significant differences between patients with dementia and patients without dementia in PD, DM, PD-DM groups were assessed by χ2-test or Mann-Whitney U tests; P-values<0.05 were considered statistically significant.

### Multivariable logistic regression analysis of risk factors of dementia in PD and PD-DM patients and the interaction of risk factors with age groups over and less than or equal to 70 years

Our study showed that lower FBG (<5mmol/L, OR=4.380; 95%CI: 1.748-10.975; p=0.002), higher HCY (>15*μ*mol/L, OR=3.131; 95%CI: 1.243-7.888; p=0.015) and hyperlipidemia (OR=3.075; 95%CI: 1.142-8.277; p=0.026) and increased age (OR=1.043; 95%CI: 1.003-1.084; p=0.034) were the most significant risk factors associated with PDD. Elevated level of Cys C (>0.95mg/L, OR=4.413; 95%CI: 1.606-12.124; p=0.004) and hyperlipidemia (OR=4.030; 95%CI: 1.289-12.605; p=0.017) were significantly associated with dementia in PD patients less than 70 years old, whereas lower FBG (<5mmol/L, OR=7.375; 95%CI: 1.689-32.198; p=0.008) was significantly associated with dementia in PD patients over 70 years old (Table 3A and Supplementary Table 3).

**Table 3A:** Multivariable logistic regression analysis for risk factors of dementia in PD patients and the interaction of risk factors with age groups.

**Variables**	**Univariate**			Multivariate Model*			**Interaction**
	**OR (95%CI)**	**p**		**Adjusted OR (95% CI)**	**p**		**p**
**Age**	**1.035(1.000,1.071)**	**0.050**		**1.043(1.003,1.084)**	**0.034**		-
**FBG<5.00 (mmol/L)**	**2.704(1.171,6.241)**	**0.020**		**4.380(1.748,10.975)**	**0.002**		
**Age≤70 (years)**	**2.609 (1.602,4.247)**	**<0.001**		2.216(0.720,6.825)	0.166		0.204
**Age>70 (years)**	**0.383(0.235,0.624)**	**<0.001**		**7.375(1.689,32.198)**	**0.008**		
**HCY>15.00 (*μ*mol/L)**	**2.684(1.156,6.231)**	**0.022**		**3.131(1.243,7.888)**	**0.015**		
**Age≤70 (years)**	**0.468(0.254,0.864)**	**0.015**		2.776(0.751,10.258)	0.126		0.709
**Age>70 (years)**	**2.135(1.158,3.935)**	**0.015**		4.005(0.977,16.420)	0.054		
**Hyperlipidemia**	2.448(0.980,6.119)	0.055		**3.075(1.142,8.277)**	**0.026**		
**Age≤70 (years)**	1.216(0.583,2.536)	0.603		**4.030(1.289,12.605)**	**0.017**		0.271
**Age>70 (years)**	0.823(0.394,1.716)	0.603		0.957(0.097,9.469)	0.970		
**Cys C>0.95 (mg/L)**	**2.122(1.008,4.468)**	**0.048**		2.157(0.979,4.752)	0.056		
**Age≤70 (years)**	0.840(0.517,1.366)	0.483		**4.413(1.606,12.124)**	**0.004**		**0.048**
**Age>70 (years)**	1.190(0.732,1.934)	0.483		0.710(0.158,3.193)	0.655		

* We performed the logistic regression models considering (a) all patients, (b) less than or equal to 70 years and (c) patients over 70 years. PD- patients with Parkinson disease without type 2 diabetes mellitus; OR- odds ratio; CI- confidence interval; FBG- fasting blood glucose; HCY- homocysteine; Cys C- Cystatin C.

In addition, a lower LDL-C (<2mmol/L, OR=4.499; 95%CI: 1.568-12.909; p=0.005) and higher fibrinogen (>4g/L, OR=4.066; 95%CI: 1.467-11.274; p=0.007) were the most significant risk factors associated with PD-DMD. Decreased LDL-C (<2mmol/L, OR=9.197; 95%CI: 2.342-36.119; p=0.001) and SAE (OR=5.389; 95%CI: 1.270-22.875; p=0.022) were significantly associated with dementia in PD-DM patients over 70 years old (Table 3B). Those variables that were not significantly associated with PDD and PD-DMD are listed in Table 3A and Table 3B. These risk factors were also found to be associated with MMSE score using the multiple linear regression analysis (Supplementary Table 1 and Supplementary File). However, all of these risk factors did not show significant interactions (p>0.05) with these two age groups except Cys C (>0.95mg/L).

**Table 3B:** Multivariable logistic regression analysis for risk factors of dementia in PD-DM patients and the interaction of risk factors with age groups.

**Variables**	**Univariate**			Multivariate Model*			**Interaction**
	**OR (95%CI)**	**p**		**Adjusted OR (95% CI)**	**p**		**p**
**Age**	**1.069(1.022,1.119)**	**0.004**		1.037(0.965,1.114)	0.325		-
**Fibrinogen>4.00 (g/L)**	**2.896(1.327,6.320)**	**0.008**		**4.066(1.467,11.274)**	**0.007**		
**Age≤70 (years)**	0.609(0.319,1.164)	0.134		4.805(0.397,58.185)	0.217		0.757
**Age>70 (years)**	1.641(0.859,3.136)	0.134		**3.109(0.955,10.128)**	**0.060**		
**LDL-C<2.00 (mmol/L)**	**3.869(1.710,8.754)**	**0.001**		**4.499(1.568,12.909)**	**0.005**		
**Age≤70 (years)**	**0.323(0.150,0.694)**	**0.004**		0.702(0.019,26.634)	0.849		0.194
**Age>70 (years)**	**3.100(1.441,6.668)**	**0.004**		**9.197(2.342,36.119)**	**0.001**		
**SAE**	**3.923(1.788,8.606)**	**0.001**		2.570(0.798,8.277)	0.114		
**Age≤70 (years)**	**0.449(0.234,0.860)**	**0.016**		3.631(0.136,97.022)	0.442		0.829
**Age>70 (years)**	**2.227(1.162,4.268)**	**0.016**		**5.389(1.270,22.875)**	**0.022**		
**WMLs**	**2.476(1.141,5.370)**	**0.022**		2.900(0.804,10.456)	0.104		
**Age≤70 (years)**	0.882(0.498,1.564)	0.668		3.808(0.114,127.097)	0.455		0.773
**Age>70 (years)**	1.133(0.639,2.009)	0.668		2.179(0.511,9.296)	0.293		
**Brain infarction**	**5.926(2.180,16.107)**	**<0.001**		2.101(0.598,7.382)	0.247		
**Age≤70 (years)**	0.584(0.334,1.024)	0.061		1.647(0.096,28.102)	0.730		0.862
**Age>70 (years)**	1.711(0.976,2.998)	0.061		2.183(0.510,9.339)	0.293		
**Anxiety or depression**	**2.250(1.031,4.908)**	**0.042**		1.898(0.682,5.279)	0.220		
**Age≤70 (years)**	**1.849(1.054,3.245)**	**0.032**		1.144(0.063,20.765)	0.927		0.723
**Age>70 (years)**	**0.541(0.308,0.949)**	**0.032**		2.011(0.636,6.359)	0.234		
**Neutrophil**	**1.057(1.019,1.097)**	**0.003**		1.102(0.920,1.320)	0.293		
**Age≤70 (years)**	**0.942(0.914,0.971)**	**<0.001**		1.456(0.830,2.552)	0.190		0.408
**Age>70 (years)**	**1.061(1.029,1.094)**	**<0.001**		1.129(0.911,1.400)	0.268		
**Lymphocyte**	**0.945(0.906,0.986)**	**0.009**		1.097(0.894,1.346)	0.374		
**Age≤70 (years)**	**1.077(1.040,1.116)**	**<0.001**		1.335(0.741,2.402)	0.336		0.664
**Age>70 (years)**	**0.928(0.896,0.962)**	**<0.001**		1.159(0.903,1.487)	0.248		

* We performed the logistic regression models considering (a) all patients, (b) less than or equal to 70 years and (c) patients over 70 years. PD-DM- patients with Parkinson disease and type 2 diabetes mellitus; OR- odds ratio; CI- confidence interval; LDL-C- low density lipoprotein cholesterol; SAE- subcortical arteriosclerotic encephalopathy; WMLs- White matter lesions.

### ROC curves of risk factors in PD-DM patients with dementia

**Figure 3:**
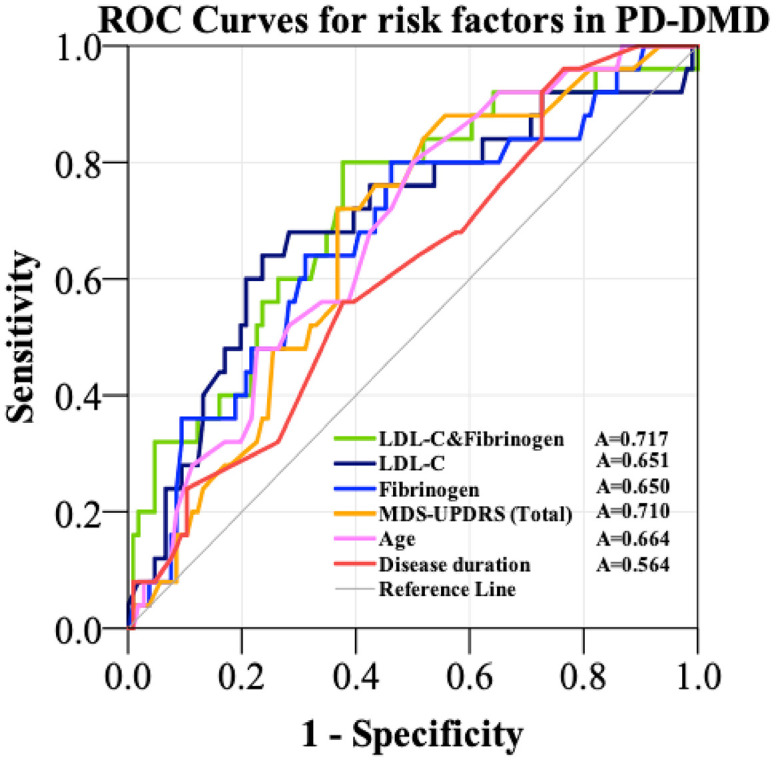
**ROC curves to evaluate the utility of novel risk factors (LDL-C and Fibrinogen, LDL-C, Fibrinogen) and traditional diagnostic factors (MDS-UPDRS, age, disease duration) for the discrimination of PD-DM patients with dementia from PD-DM patients without dementia.** The AUC was 0.650 for fibrinogen (blue curve), 0.651 for LDL-C (black curve) and 0.717 for the combination of LDL-C and fibrinogen (green curve). The AUC was 0.564 for disease duration (red curve), 0.664 for age (purple curve) and 0.710 for MDS-UPDRS (orange curve). PD-DMD- patients with Parkinson’s Disease and type 2 diabetes mellitus and dementia; LDL-C- low density lipoprotein cholesterol; A- area under the curve; MDS-UPDRS- Movement Disorder Society–Unified Parkinson’s Disease Rating Scale; ROC- Receiver Operating Characteristic.

ROC curves were constructed to explore which factor could provide useful discrimination between PD-DM patients with dementia and without dementia. ROC curves for disease duration, age and MDS-UPDRS (total) revealed that the AUC was 0.564 (95%CI: 0.458-0.669, p=0.262), 0.664 (95%CI: 0.568-0.760, p=0.004) and 0.710 (95%CI: 0.622-0.798, p<0.001). ROC curves for fibrinogen and LDL-C analysis revealed AUC values of 0.650 (95%CI: 0.537-0.763, p=0.015) and 0.651 (95%CI: 0.534-0.769, p=0.009), respectively. However, the combination of fibrinogen and LDL-C increased the AUC to 0.717 (95%CI: 0.606-0.828, p=0.001), with a sensitivity of 80.0% and a specificity of 62.8% at a cutoff value of 0.155 on the predicted risk algorithm (Table 4 and Figure 3).

**Table 4:** ROC curves for the traditional diagnostic and novel risk factors in the diagnosis of PD-DM patients with dementia.

**Variable**	**Traditional diagnostic factors**				**Novel risk factors**		
	**Disease duration**	**Age**	**MDS-UPDRS (Total)**		**Fibrinogen (g/L)**	**LDL-C (mmol/L)**	**Combination (LDL-C & Fibrinogen)**
**AUC**	**0.564**	**0.664**	**0.710**		**0.650**	**0.651**	**0.717**
**Cut-off value**	0.170	0.257	0.375		0.308	0.335	0.428
**P value**	0.262	**0.004**	**<0.001**		**0.015**	**0.009**	**0.001**
**95%CI**	0.458-0.669	0.568-0.760	0.622-0.798		0.537-0.763	0.534-0.769	0.606-0.828
**Sensitivity**	**0.903**	**0.806**	**0.742**		**0.808**	0.552	**0.800**
**Specificity**	0.267	0.451	**0.633**		0.500	**0.783**	**0.628**

PD-DM- patients with Parkinson’s disease and type 2 diabetes mellitus; LDL-C- low density lipoprotein cholesterol; AUC- area under the curve; MDS-UPDRS- Movement Disorder Society–Unified Parkinson’s Disease Rating Scale; CI- confidence interval; ROC- receiver operating characteristic.

## DISCUSSION

We conducted the first comparative study between PD-DMD and PDD patients. We highlighted that PD-DMD patients exhibited more severe motor and nonmotor symptoms and vascular inflammation derangements than PDD patients. Vascular inflammatory risk factors such as LDL-C and fibrinogen were more prevalently associated with PD-DMD than PDD and might have prognostic and treatment implications (Supplementary Table 5). We identified LDL-C<2.00 (mmol/L) and fibrinogen>4.00 (g/L) as the most significant risk factors for PD-DMD and FBG<5.00 (mmol/L), HCY>15.00 (*μ*mol/L) and hyperlipidemia for PDD patients. Preventing a lower LDL-C<2.00 (mmol/L) and a higher fibrinogen>4.00 (g/L) might be effective to reduce dementia in PD-DM patients.

There are possible explanations for the relative difference in the association of the vascular and inflammatory risk factors between PD-DMD and PD-DM without dementia. PD-DM patients have been shown to be more prone to develop cognitive impairment in the course of their disease than patients with PD but without DM [14]. It is possible that upregulation of vascular inflammatory factors in comorbid PD and DM, exacerbates brain toxicity, leading to the development of dementia [22]. This observation is consistent with emerging evidence that interactions between several vascular inflammatory risk factors are linked to target organ damage [23]. Vascular inflammation may exacerbate the progression of PD-DM patients, including greater fragmentation of capillaries and chronic inflammatory damage to the capillary network in multiple brain regions, particularly in the frontal cortex, hippocampus, temporal lobe and prefrontal lobe. These cognition-related cerebral damages caused by vascular inflammation will lead to more accelerated decline of cognition in PD-DM patients [24–26]. Thus, treatments that regulate vascular inflammation can potentially improve vascular remodeling in the brain and provide a novel target to ameliorate the disease burden in PD-DM patients [11, 27].

A previous study that showed a high level of Cys C and HCY in PD, and their levels were higher in PD patients with dementia than in those without dementia [28]. This is consistent with our findings that high levels of homocysteine (HCY>15 *μ*mol/L) and elevated Cys C (Cys C>0.95 mg/L) are the important risk factors for PDD patients (Table 3A). The dysregulation of iron metabolism associated with cellular damage (MCV of erythrocyte increased) and oxidative stress are common in PDD [29]. Furthermore, hyperlipidemia, especially hypercholesterolemia, is a recognized risk factor in cardiovascular disease, and the total lifelong cholesterol burden contributes to the risk, which might also lead to dementia in PD [22].

Interestingly, we identified more brain infarctions in PD-DMD patients than in PDD patients; SAE was more significantly associated with dementia in PD-DM patients over 70 years old (Figure 2 and Table 3B). This finding is similar to that of previous observations relating such brain imaging changes to cognitive impairment in PD [6, 12, 30, 31]. This finding further indicates that cerebrovascular lesions or degeneration may be more prevalent in PD-DMD patients than in PDD patients and may contribute more to dementia with comorbid DM and PD patients. Thus, treatments that prevent vascular degeneration or ameliorate brain infarctions may improve vascular remodeling in the brain and provide a novel target to ameliorate the cognitive burden in patients with PD-DMD.

Fibrinogen is highly expressed in PD-DMD patients and is the most significant risk factor for dementia in PD-DM patients (Table 3B). Fibrinogen is a vascular inflammatory mediator that has recently been shown to drive the neurodegenerative process in dementia, cerebrovascular stroke and PD. It is postulated that this maybe via the activation of CNS vascular inflammation, the dysregulation of microcirculatory function and the disruption of the BBB and neurovascular units (NVUs) [32, 33]. Investigators have shown that following BBB disruption, fibrin and fibrinogen interact with receptors on nervous system cells to activate downstream signaling, regulate basic cellular functions and influence inflammatory and neurodegenerative processes in disease [32]. Fibrin deposition precedes neuronal degenerative changes and behavioral deficits in pericyte-mutant mice, suggesting that fibrin entry into the CNS is a critical factor that initiates or potentiates neurodegenerative processes after vascular disruption [32].

Cholesterol is a major component of the brain, and a lower cholesterol level in elderly individuals is associated with cerebral atrophy, a typical anatomic syndrome of dementia [34, 35]. We showed that a low level of LDL-C (<2 mmol/L, Table 3B) may increase dementia risk, especially in PD-DM patients over 70 years. This could be due to aggravation of cerebral atrophy, malnutrition and the reduction in neuron impairment or facilitation of the compensatory repair of injured neurons.

After selecting the potential risk factors including fibrinogen and LDL for dementia in PD-DM patients using logistic regression, we further explored their utility using ROC analysis to compare the discriminatory ability of predictors of dementia in PD-DM patients. Conventionally, it is accepted that the AUC in ROC analysis should be >0.7 to be of clinical value for screening. During the discovery phase, the AUC for the combination of fibrinogen and LDL-C proved to be better than using either alone (with the AUC increasing from 0.650 and 0.651 for either alone to 0.717, with a sensitivity of 80.0% and a specificity of 62.8%) or traditional diagnostic factors (disease duration, age, MDS-UPDRS) in the prediction of dementia.

Our study has some inherent limitations. The hospital-based setting of our study may have resulted in a selection bias for structural brain imaging based on clinical decisions, which is likely to overestimate the general prevalence of cerebrovascular disease in patients with PD and DM. The diagnosis of PD and PD-DM in our cohort was based on clinical diagnostic criteria, with no post mortem confirmation [31]. Our study has certain strengths. We have investigated a large number of patients with comorbid DM and PD. This is the first comparative study on vascular, inflammatory and metabolic risk factors on PDD and PD-DMD, and the inclusion of PD patients without DM and DM patients without PD as control groups is a major strength.

## CONCLUSION

PD-DMD patients impose considerable public health burdens and are commonly encountered in clinical practice. The interactions between PD, cognitive dysfunction and diabetes mellitus are likely to be complex. Our study identified controllable clinical factors, including LDL-C and fibrinogen, as the most significant risk factors for PD-DMD patients; they were more prevalently associated with PD-DMD than PDD patients. As these vascular, inflammatory and metabolic risk factors are modifiable, monitoring and corrections of these factors could potentially improve clinical care and provide a new treatment paradigm for PD and PD-DM patients. Furthermore, our findings can also lead to identification of novel therapeutic targets for preventing cognitive impairment in PD and PD-DM patients.

## MATERIALS AND METHODS

### Population

We recruited a total of 928 patients from outpatient clinics. A total of 215 PD-DM patients (including 31 PD-DM with dementia, PD-DMD), 341 PD patients without DM (including 31 PDD) and 372 DM patients without PD (including 35 DM with dementia, DMD) were included for this comparative study. PD patients without DM and DM patients without PD were set as controls. All PD and PD-DM patients underwent a standardized neurological examination by two movement disorder specialists in a blinded manner. The PD patients recruited in this present study (Figure 1) satisfied the 2015 Movement Disorder Society criteria for the diagnosis of idiopathic PD [36]. The diagnosis of DM was made by 2 physicians according to the following diagnostic criteria: a tested fasting glucose level higher than 7.0 mmol/L, a 2-h postprandial glucose level higher than 11.1 mmol/L, or pre-diagnosed type 2 diabetes [37]. Those who failed to meet the 2015 Movement Disorder Society criteria for PD or the diagnostic criteria for DM or were younger than 18 years (Figure 1) were excluded. Patients with fever (n=163), infection (n=150), severe traumatic brain injury (n=657), sever comorbid illness (n=500), tumor (n=2000) which might significantly impact movement and cognition, other degenerative forms of or drug-induced parkinsonism (n=100) and symmetrical lower body parkinsonism attributable to significant cerebrovascular disease (n=1400) were also excluded. Other information can be found in the Supplementary Files.

### Study design and ethics statement

The data were retrospectively collected from patients with PD, DM and PD-DM with or without dementia at Zhujiang Hospital of Southern Medical University, Guangzhou, Guangdong, China over a 6-year period (January 2013 to May 2019). This study was approved by the Human Research Committee at Zhujiang Hospital of Southern Medical University (No: 2019-KY-030-02). Written informed consent was obtained from all participants or their legal guardians.

Experienced neurologists were recruited to perform the evaluations and completed the neurological examinations for all subjects. All subjects completed the following battery of standard assessment measures: a standard demography form, the Movement Disorder Society–Unified Parkinson’s Disease Rating Scale (MDS-UPDRS) [38], and the modified Hoehn and Yahr (H&Y) staging scale. The UPDRS (I) ‘mentation’ and UPDRS (II) ‘daily life’ subscales were used to evaluate psychiatric dysfunction and disease severity. The UPDRS (III) ‘motor’ and H&Y subscales were used to evaluate motor dysfunction and disease severity. The degree of nonmotor symptoms (NMSs) in every patient was measured by the NMS scale (NMSS) [39]. Cognitive abilities were evaluated with the Mini Mental State Examination (MMSE) [40] and the Montreal Cognitive Assessment (MoCA). MoCA scores were adjusted for years of education. Predetermined diagnostic cut-offs were used to categorize cases into dementia (21 or less with functional impairment) to reflect the core criteria for PD dementia (PDD) with or without DM as defined by the Movement Disorder Society Task Force [41, 42]. All scales were available and validated. Patients were considered to present with anxiety or depression, brain infarction, WMLs, SAE or hyperlipidemia if there was a self-reported or doctor-diagnosed history of these conditions or if they were using disease-related medications. When WMLs result from hypoxic-ischemic brain lesions and lead to cognitive impairment, this complex disorder is characterized as SAE [17]. Individuals were considered to have hyperlipidemia at screening if they had a total cholesterol level of more than 6.2 mmol/L (240 mg/dL), serum triglycerides concentrations more than 2.3 mmol/L (200 mg/dL), serum LDL-C concentrations more than 4.1 mmol/L (160 mg/dL) or serum HDL-C concentrations less than 1.0 mmol/L (40 mg/dL) [43–47]. Hypoglycemia was defined in subjects with a plasma glucose of ≤3.9mmol/L and/or self-reported probable hypoglycemic symptoms [48].

In addition, routine examinations and imaging scans during hospitalization were completed. Venous blood samples from all participants were collected into Ethylene Diamine Tetraacetic Acid (EDTA) tubes by trained nurses in the morning. Blood samples were sent to the clinical pathology department immediately. The serum levels of all biochemical factors were measured by clinical pathologists. The serum hsCRP, Cys C and albumin were measured by immunoturbidimetric assay (Orion Diagnostica, Espoo, Finland); serum LDL-C and D-dimer were measured with direct enzymatic methods; serum AST and ALT were measured using the International Federation of Clinical Chemistry (IFCC) method. Serum HCY levels were measured using HCY reagent (Maccura Biotechnology Co., Ltd., Chengdu, China) through enzymatic cycling assay by Hitachi Automatic Analyzer 7600–210 (Hitachi, Tokyo, Japan). Serum fibrinogen levels were examined with commercial kits following the manufacturer's instructions by a coagulometer (SC40 semi-automatic coagulation analyzer, Taizhou Steellex Biotechnology Co., LTD., China). All participants underwent T1-weighted, T2-weighted, and fluid-attenuated inversion recovery (FLAIR) imaging using a 3.0-T MRI scanner (Philips Healthcare, Andover, MA). Two neuroradiologists blinded to the clinical information of patients independently rated periventricular white matter hyperintensities and deep white matter hyperintensities using a modified Fazekas scale to assess the degree of severity of WMLs on T2 MR images [24, 49]. The enrolled patients were divided into 3 groups according to age (Table 1): less than 55, 55 to 70 and more than 70 years old [50–53]. The relative variables [LDL-C, D-Dimer, fibrinogen, hypersensitive C-reactive protein (hs-CRP), aspartate transaminase (AST), albumin, calcium, mean corpuscular volume (MCV), HCY, Cys C, FBG] were categorized according to the normal reference range, and we considered the levels of biochemical indicators as the commonly used thresholds in this study (Supplementary Files).

### Statistical analyses

All continuous variables, including age, PD duration, MDS-UPDRS, H&Y, NMSS, MMSE, and MoCA scores; vascular and inflammatory risk factors, including systolic blood pressure (SBP), diastolic blood pressure (DBP), body mass index (BMI), and LDL-C, D-dimer, fibrinogen, WBC, and hs-CRP levels; and metabolic-related risk mediators, including AST, alanine transaminase (ALT), albumin, calcium, potassium, MCV, HCY, Cys C, FBG and glycated hemoglobin (HbA1c) levels, are presented as the median (interquartile range, IQR) as they were not normally distributed. All categorical variables including male gender; smoking history; drinking history; drug use, including atorvastatin, metformin, insulin, acarbose, levodopa and benserazide (L-Dopa); anxiety or depression; vascular and inflammatory risk factors including hyperlipidemia, brain infarction, SAE, WMLs, lymphocytes, and neutrophils; and metabolic risk factors, including hyperhomocysteinemia, are presented as the count (proportion). We selected these most common used lipid-lowering drug (atorvastatin), hypoglycemic drugs (metformin and acarbose) and drug for Parkinson’s disease (L-Dopa) in clinics as covariates to exclude their effects on dementia. The statistically significant differences between patients with dementia and patients without dementia in the PD, DM, and PD-DM groups were assessed by the χ2-test or Mann-Whitney U tests. P-values<0.05 were considered statistically significant, but when multiple testing was performed, the Bonferroni method was used to adjust the significance level. If statistically significant, continuous variables were assessed by Kruskal–Wallis test (nonparametric one-way analysis of variance, ANOVA) followed by post-hoc analysis with Bonferroni adjustment to compare differences among PDD, DMD and PD-DMD groups and were prior adjusted for age using multivariate linear regression analyses. Categorical parameters were analyzed using the χ2-test with Bonferroni adjustment for multiple testing. Mann-Whitney U test were used to compare differences between PDD and PD-DMD groups. Binary logistic regression was applied to explore the potential risk factors and in PDD and PD-DMD patients. The presence or absence of dementia was used as a dependent variable. All vascular inflammatory and metabolic risk factors demonstrating significant differences between patients with and without dementia (selection criterion p<0.05) were included in the binary logistic regression analysis with enter selection (Table 3). The results are presented as odds ratios (ORs) and 95% confidence intervals (CIs). Since several lines of evidence showed that age around 70 years would be critical age for dementia in PD and PD-DM patients[50, 52–54], we performed the logistic regression models considering (a) all patients, (b) less than or equal to 70 years and (c) patients over 70 years. In addition, multiple linear regression (enter method) was also used to investigate whether the MMSE score was associated with the risk factors of dementia. Receiver operating characteristic (ROC) curves of logistic regression prediction models for PD-DMD were used with the risk factors (LDL-C and fibrinogen) in PD-DM patients. We also compared the accuracy, sensitivity and specificity of novel (LDL-C and fibrinogen) and traditional (disease duration, age, MDS-UPDRS) risk factors and used bootstrap validation with 1000 repeats (Supplementary Figure 1). All statistical procedures were conducted using Statistical Package for the Social Sciences (SPSS) version 20.0 and GraphPad Prism 7.0. P-values<0.05 were considered statistically significant.

## Supplementary Material

Supplementary Materials

Supplementary Figure 1

Supplementary Tables
