# Supplementary material for: Vascular, inflammatory and metabolic risk factors in relation to dementia in Parkinson’s disease patients with type 2 diabetes mellitus
Source: Aging (Albany NY). 2020 Aug 15;12(15):15682–704. doi: 10.18632/aging.103776 (PMC7467390; doi:10.18632/aging.103776)
Supplement: Supplementary Materials [file aging-12-103776-s001..pdf]

SUPPLEMENTARY MATERIALS

Supplementary Methods

Personal history of smoking behavior was calculated by "pack/years" quantifying the packs smoked per day multiplied by years as a smoker, with the threshold was set to 15. Drinking was defined as an average of alcoholic drink (≥ 50 ml) at least once per week lasting more than half a year. Body-mass index (BMI) was calculated as body weight (kg) divided by heights squared (m^2).

The following variables were categorized according to the normal reference range [1–11] and we considered the following levels of biochemical indicators as the commonly used thresholds in this study: the low LDL level as less than 2.00 mmol/L [1], the high level of D-Dimer as more than 0.50mg/L [2], the high level of fibrinogen as more than 4.00g/L [3], the high level of hypersensitive C-reactive protein (hs-CRP) as more than 3.00mg/L [4], the high level of aspartate transaminase (AST) as more than 40IU/L [5], the low level of albumin as less than 35.00g/L [6], the low level of calcium as less than 2.10mmol/L [7], the high level of mean corpuscular volume (MCV), as more than 90.00fL [8], the high level of HCY as more than 15 mmol/L (the cutoff for hyperhomocysteinemia) [9], the high level of Cys C as more than 0.95mg/L [10] and the low level of fasting blood glucose (FBG) as less than 5.00mmol/L [11].

Supplementary Results

Multiple linear regression analysis for the association of risk factors of dementia and MMSE score in PD, DM and PD-DM patients

Using multiple linear regression analysis, we found that a lower FBG (<5.00 mmol/L, B= -1.052, SE=0.433, β=-0.126, p=0.016), higher HCY (>15.00 μmol/L, B= -1.336, SE=0.595, β= -0.115, p=0.026), and Cys C (>0.95 mg/L, B= -0.908, SE=0.452, β= -0.103, p=0.046), and age (B=-0.140, SE=0.019, β= -0.376, p<0.001) were associated with lower MMSE score. No significant association was observed between MMSE score and hyperlipidemia (B= -1.232, SE=0.641, β=-0.098, p=0.056) in PD patients (adjusted R^2=0.173, F=14.469, p<0.001). In addition, higher fibrinogen (>4.00 g/L, B= -1.857, SE=0.663, β= -0.174, p=0.006), lower LDL-C (<2.00 mmol/L, B= -1.876, SE=0.674, β= -0.173, p=0.006), age (B= -0.171, SE=0.032, β= -0.342, p<0.001) and SAE (B= -2.196, SE=0.640, β= -0.216, p<0.001) were associated with lower MMSE score in PD-DM patients (adjusted R^2=0.275, F=19.383, p<0.001). A higher AST (>40 IU/L, B= -3.278, SE=1.023, β= -0.141, p=0.001) and Cys C (>0.95 mg/L, B= -1.878, SE=0.690, β= -0.119, p=0.007), anxiety or depression (B= -1.579, SE=0.531, β= -0.135, p=0.003), male gender (B= -1.182, SE=0.398, β= -0.131, p=0.003) and age (B= -0.163, SE=0.015, β= -0.481, p<0.001) were associated with lower MMSE score in DM patients (adjusted R^2=0.310, F=34.277, p<0.001) (Supplementary Table 1). These observations are consistent with the results of multivariable logistic regression analysis for risk factors in dementia in PD, DM and PD-DM patients (Table 3A, Table 3B, Supplementary Table 4).

Supplementary References

1. Lin Q, Huang WQ, Ma QL, Lu CX, Tong SJ, Ye JH, Lin HN, Gu L, Yi KH, Cai LL, Tzeng CM. Incidence and risk factors of leukoaraiosis from 4683 hospitalized patients: a cross-sectional study. Medicine (Baltimore). 2017; 96:e7682. https://doi.org/10.1097/MD.0000000000007682 PMID:28953609

2. Hiltunen S, Putaala J, Haapaniemi E, Salonen O, Tatlisumak T. D-dimer and clinicoradiologic features in cerebral venous thrombosis. J Neurol Sci. 2013; 327:12–14. https://doi.org/10.1016/j.jns.2013.01.033 PMID:23434247

3. McQuilten ZK, Wood EM, Bailey M, Cameron PA, Cooper DJ. Fibrinogen is an independent predictor of mortality in major trauma patients: a five-year statewide cohort study. Injury. 2017; 48:1074–81. https://doi.org/10.1016/j.injury.2016.11.021 PMID:28190583

4. Fernandes RA, Ritti-Dias RM, Balagopal PB, Conceição RD, Santos RD, Cucato GG, Bittencourt MS. Self-initiated physical activity is associated with high sensitivity c-reactive protein: a longitudinal study in 5,030 adults. Atherosclerosis. 2018; 273:131–35. https://doi.org/10.1016/j.atherosclerosis.2018.02.011 PMID:29544861

5. Larrieta-Carrasco E, Flores YN, Macías-Kauffer LR, Ramírez-Palacios P, Quiterio M, Ramírez-Salazar EG, León-Mimila P, Rivera-Paredez B, Cabrera-Álvarez G, Canizales-Quinteros S, Zhang ZF, López-Pérez TV, Salmerón J, Velázquez-Cruz R. Genetic variants in COL13A1, ADIPOQ and SAMM50, in addition to the PNPLA3 gene, confer susceptibility to elevated transaminase levels in an admixed mexican population. Exp Mol Pathol. 2018; 104:50–58. https://doi.org/10.1016/j.yexmp.2018.01.001 PMID:29307798

6. Loncar Y, Lefevre T, Nafteux L, Genser L, Manceau G, Lemoine L, Vaillant JC, Eyraud D. Preoperative nutrition forseverely malnourished patients in digestive surgery: a retrospective study. J Visc Surg. 2020; 157:107–16. https://doi.org/10.1016/j.jviscsurg.2019.07.007 PMID:31366442

7. Li D, Zhang L, Zuo L, Jin CG, Li WG, Chen JB. Association of CKD-MBD markers with all-cause mortality in prevalent hemodialysis patients: a cohort study in beijing. PLoS One. 2017; 12:e0168537. https://doi.org/10.1371/journal.pone.0168537 PMID:28045985

8. Papageorgiou A, Ziakas PD, Tzioufas AG, Voulgarelis M. Indications for bone marrow examination in autoimmune disorders with concurrent haematologic alterations. Clin Exp Rheumatol. 2013; 31:76–83. PMID:23010147

9. Licking N, Murchison C, Cholerton B, Zabetian CP, Hu SC, Montine TJ, Peterson-Hiller AL, Chung KA, Edwards K, Leverenz JB, Quinn JF. Homocysteine and cognitive function in Parkinson’s disease. Parkinsonism Relat Disord. 2017; 44:1–5. https://doi.org/10.1016/j.parkreldis.2017.08.005 PMID:28807493

10. García Acuña JM, González-Babarro E, Grigorian Shamagian L, Peña-Gil C, Vidal Pérez R, López-Lago AM, Gutiérrez Feijoó M, González-Juanatey JR. Cystatin C provides more information than other renal function parameters for stratifying risk in patients with acute coronary syndrome. Rev Esp Cardiol. 2009; 62:510–19. https://doi.org/10.1016/s1885-5857(09)71833-x PMID:19406065

11. Xu RB, Kong X, Xu BP, Song Y, Ji M, Zhao M, Huang X, Li P, Cheng X, Chen F, Zhang Y, Tang G, Qin X, et al. Longitudinal association between fasting blood glucose concentrations and first stroke in hypertensive adults in China: effect of folic acid intervention. Am J Clin Nutr. 2017; 105:564–70. https://doi.org/10.3945/ajcn.116.145656 PMID:28122783
