# Supplementary material for: Vascular, inflammatory and metabolic risk factors in relation to dementia in Parkinson’s disease patients with type 2 diabetes mellitus
Source: Aging (Albany NY). 2020 Aug 15;12(15):15682–704. doi: 10.18632/aging.103776 (PMC7467390; doi:10.18632/aging.103776)
Supplement: Supplementary Figure 1 [file aging-12-103776-s002..pdf]

SUPPLEMENTARY FIGURE

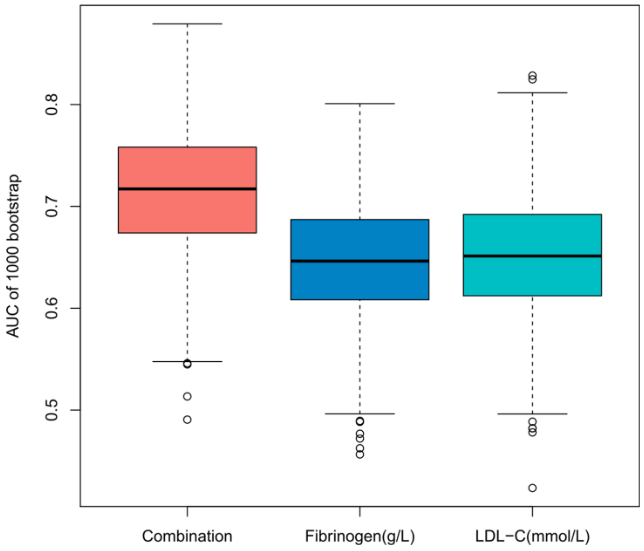

Supplementary Figure 1. The AUC distributions of 1000 bootstrap samples. The bias-corrected and accelerated (BCA) bootstrap 95% confidence interval of AUC for Fibrinogen(g/L), LDL-C(mmol/L), and their combination are 0.648 [BCA 95%CI: 0.537~0.756], 0.655 [BCA 95%CI: 0.516~0.760], and 0.716 [BCA 95%CI: 0.595~0.818]. AUC- area under the curve; LDL-C- low density lipoprotein cholesterol.
