# Supplementary material for: Vascular, inflammatory and metabolic risk factors in relation to dementia in Parkinson’s disease patients with type 2 diabetes mellitus
Source: Aging (Albany NY). 2020 Aug 15;12(15):15682–704. doi: 10.18632/aging.103776 (PMC7467390; doi:10.18632/aging.103776)
Supplement: Supplementary Tables [file aging-12-103776-s003..pdf]

SUPPLEMENTARY TABLES

Supplementary Table 1. Association of the risk factors of dementia and MMSE score in PD, DM and PD-DM patients.

Model	Predictor variables	B	SE	*β*	p	R^2	Adjusted R^2	F	p
PD	FBG<5.00 (mmol/L)	-1.052	0.433	-0.126	**0.016**	0.185	0.173	14.469	<0.001
	HCY>15.00 (*μ*mol/L)	-1.336	0.595	-0.115	**0.026**				
	Age	-0.140	0.019	-0.376	**<0.001**				
	Cys C>0.95 (mg/L)	-0.908	0.452	-0.103	**0.046**				
	Hyperlipidemia	-1.232	0.641	-0.098	0.056				
PD-DM	Fibrinogen>4.00 (g/L)	-1.857	0.663	-0.174	**0.006**	0.290	0.275	19.383	<0.001
	LDL-C<2.00 (mmol/L)	-1.876	0.674	-0.173	**0.006**				
	Age	-0.171	0.032	-0.342	**<0.001**				
	SAE	-2.196	0.640	-0.216	**0.001**				
DM	AST>40 (IU/L)	-3.278	1.023	-0.141	**0.001**	0.319	0.310	34.277	<0.001
	Cys C>0.95 (mg/L)	-1.878	0.690	-0.119	**0.007**				
	Anxiety or depression	-1.579	0.531	-0.135	**0.003**				
	Male	-1.182	0.398	-0.131	**0.003**				
	Age	-0.163	0.015	-0.481	**<0.001**				

B- Unstandardized coefficient; *β*- Standardized coefficient; R^2 represents the model explanatory power with according standardized β weights, showing the contribution of each independent variable to the proposed model; SE- Standard Error; MMSE- mini mental state examination; PD- patients with Parkinson disease without type 2 diabetes mellitus; PD-DM- patients with Parkinson disease and type 2 diabetes mellitus; DM- type 2 diabetes mellitus without Parkinson disease; FBG- Fasting blood glucose; HCY- homocysteine; Cystatin C- Cys C; LDL-C- low density lipoprotein cholesterol; SAE- subcortical arteriosclerotic encephalopathy; AST- Aspartate transaminase.

Supplementary Table 2. Differences of risk factors among PDD, DMD and PD-DMD groups.

Clinical variables	PDD (n=31)	DMD (n=35)	PD-DMD (n=31)	p*	Multiple comparisons **
**Age, years**	69.0(59.0,79.0)	68.0(57.0,74.0)	79.0(72.0,84.0)	**<0.001**	**PD-DMD>PDD=DMD**
<55, n (%)	3(9.68)	7(20.00)	0	-	-
55–70, n (%)	16(51.61)	14(40.00)	5(16.13)	-	-
>70, n (%)	12 (38.71)	14(40.00)	26(83.87)	**<0.001**	**PD-DMD>PDD=DMD**
Male, n (%)	18(58.06)	27(77.14)	16(51.61)	0.080	-
**PD duration, month**	60(24,84)	-	36(12,72)	-	
**Dementia, n (%)**	31 (100.00)	35 (100.00)	31 (100.00)	0.094	-
**Use of drugs, n (%)**					
Atorvastatin	6(19.35)	14(40.00)	20(64.52)	**0.001**	**PD-DMD>PDD**
Metformin	-	14(40.00)	9(29.03)	-	-
Insulin	-	8(22.86)	14(45.16)	0.055	-
Acarbose	-	17(48.57)	18(58.06)	0.441	-
L-Dopa	28(90.32)	-	26(83.87)	-	-
**MDS-UPDRS (I)**	3.0(2.0,5.0)	-	5.0(3.0,6.0)	**0.006**	**PD-DMD>PDD**
**MDS-UPDRS (II)**	8.0(6.0,11.0)	-	14.0(12.0,18.0)	**<0.001**	**PD-DMD>PDD**
**MDS-UPDRS (III)**	17.0(15.0,23.0)	-	23.0(21.0,29.0)	**<0.001**	**PD-DMD>PDD**
**MDS-UPDRS (Total)**	26.0(24.0,38.0)	-	44.0(37.0,54.0)	**<0.001**	**PD-DMD>PDD**
**H&Y**	3.0(2.0,4.0)	-	3.0(3.0,4.0)	0.072	-
**NMSS**	26.0(17.0,32.0)	-	32.0(28.0,34.0)	**0.003**	**PD-DMD>PDD**
**MMSE**	15.0(13.0,17.0)	15.0(9.0,19.0)	13.0(11.0,17.0)	0.613	-
**MoCA**	12.0(9.0,14.0)	12.0(7.0,15.0)	9.0(8.0,13.0)	0.205	-
**Anxiety or depression**	17(54.84)	13(37.14)	19(61.29)	0.124	-
**Vascular and inflammatory risk factors**					
**SBP mmHg**	120.0(118.0,135.0)	146.0(128.0,153.0)	132.0(120.0,152.0)	-	-

**DBP mmHg**	80.0(78.0,81.0)	85.0(80.0,95.0)	78.0(69.0,87.0)	-	-
**Smoking history, n (%)**	2(6.45)	7(20.00)	3(9.68)	-	-
**Drinking history, n (%)**	0	5(14.29)	1(3.23)	-	-
**BMI**	23.0(22.0,25.0)	24.0(22.0,26.0)	24(21,25)	-	-
**LDL-C (mmol/L)**	2.5(2.3,3.2)	2.7(1.9,3.1)	1.9(1.6,3.0)	0.094	-
LDL-C<2.00 (mmol/L), n (%)	2(6.45)	12(34.29)	15(48.39)	**0.001**	**PD-DMD=DMD>PDD**
**D-Dimer (mg/L)**	0.6(0.3,1.1)	0.4(0.3,0.9)	0.6(0.4,1.4)	-	-
D-Dimer>0.50 (mg/L), n (%)	10(32.26)	8(22.86)	10(32.26)	0.618	-
**Fibrinogen (g/L)**	3.5(2.8,4.0)	3.3(3.0,4.6)	4.1(3.5,5.1)	**0.012**	**PD-DMD>PDD**
Fibrinogen>4.00 (g/L), n (%)	6(19.35)	9(25.71)	15(48.39)	**0.033**	**PD-DMD>PDD**
**WBC (g/L)**	6.1(5.0,7.2)	7.3(6.0,9.5)	7.4(6.1,9.9)	-	-
**Lymphocyte (%)**	30.7(24.2,34.8)	25.3(18.6,32.1)	20.5(12.2,28.0)	**0.001**	**PD-DMD<PDD**
**Neutrophil (%)**	58.6(52.5,67.5)	64.0(58.3,70.2)	71.5(61.6,82.8)	**<0.001**	**PD-DMD>PDD**
**hs-CRP (mg/L)**	0.6(0.6,2.0)	3.6(1.5,7.1)	4.5(0.7,47.1)	0.036	-
hs-CRP>3.00 (mg/L), n (%)	2(6.45)	11(31.43)	8(25.81)	**0.039**	**DMD>PDD**
**Hyperlipidemia**	7(22.58)	3(8.57)	2(6.45)	-	-
**Brain infarction**	13(41.94)	27(77.14)	26(83.87)	**0.001**	**PD-DMD=DMD>PDD**
**SAE**	9(29.03)	5(14.29)	18(58.06)	**0.001**	**PD-DMD>DMD**
WMLs, n (%)	12(38.71)	17(48.57)	18(58.06)	0.313	-
Fazekas 1	5(16.13)	2(5.71)	2(6.45)	-	-
Fazekas 2	7(22.58)	12(34.29)	15(48.39)	0.103	-
Fazekas 3	0	3(8.57)	1(3.23)	0.322	-
**Metabolic risk factors**					
**AST>40(IU/L), n (%)**	0	5(14.29)	3(9.68)	0.091	-
**ALT**	15.0(9.0,22.0)	22.0(15.0,28.0)	14.0(11.0,30.0)	-	-
**Albumin**	40.3(38.0,41.8)	39.3(36.2,42.9)	36.9(36.0,42.1)	0.204	-
Albumin<35.00 (g/L), n (%)	2(6.45)	4(11.43)	7(22.58)	0.187	-
**Calcium (mmol/L)**	2.3(2.2,2.4)	2.2(2.2,2.3)	2.2(2.1,2.3)	0.329	-
Calcium<2.10 (mmol/L), n (%)	5(16.13)	6(17.14)	8(25.81)	0.569	-
**Potassium (mmol/L)**	3.8(3.6,4.1)	3.9(3.6,4.0)	3.8(3.4,4.3)	0.911	-
**MCV (fL)**	92.7(91.1,97.4)	87.2(83.5,93.3)	89.9(88.2,93.2)	**<0.001**	**PDD>PD-DMD=DMD**
MCV>90.00 (fL), n (%)	25(80.65)	13(37.14)	14(45.16)	**0.001**	**PDD>PD-DMD=DMD**
**HCY (*μ*mol/L)**	15.8(12.0,17.8)	11.3(8.5,15.3)	12.3(8.7,18.0)	-	-
HCY>15.00 (*μ*mol/L), n (%)	9(29.03)	5(14.29)	5(16.13)	0.270	-
**Hyperhomocysteinemia**	4(12.90)	0	1(3.23)	0.041	-
**Cys C (mg/L)**	1.1(1.0,1.2)	1.0(0.9,1.1)	1.0(0.9,1.5)	0.551	-
Cys C>0.95 (mg/L), n (%)	15(48.39)	8(22.86)	7(22.58)	0.039	-
**FBG (mmol/L)**	4.7(4.3,5.1)	7.6(5.8,11.6)	7.1(6.1,10.0)	**<0.001**	**PD-DMD=DMD>PDD**
FBG<5.00 (mmol/L), n (%)	23(74.19)	5(14.29)	1(3.23)	**<0.001**	**PD-DMD=DMD<PDD**
**HbA1c (%)**	5.7(5.2,5.9)	7.3(6.3,8.9)	6.4(6.0,7.4)	-	-

PDD- patients with Parkinson disease with dementia; DMD- type 2 diabetes mellitus with dementia; PD-DMD- patients with Parkinson disease and type 2 diabetes mellitus and dementia; L-Dopa- Levodopa and Benserazide; MDS-UPDRS- Movement Disorder Society–Unified Parkinson's Disease Rating Scale; H&Y- the modified Hoehn and Yahr staging scale; NMSS- Non-Motor Symptoms Scale for Parkinson's Disease; MMSE- mini mental state examination; MoCA- Montreal Cognitive Assessment; SBP- systolic blood pressure; DBP- diastolic blood pressure; BMI- body mass index; LDL-C- low density lipoprotein cholesterol; WBC- White blood cell count; hs- CRP- hypersensitive C-reactive protein; SAE- subcortical arteriosclerotic encephalopathy; WMLs- White matter lesions; AST- Aspartate transaminase; ALT- Alanine transaminase; MCV- mean corpuscular volume; HCY- homocysteine; Cystatin C- Cys C; FBG- Fasting blood glucose; HbA1c- glycated hemoglobin. *The Bonferroni method was used to adjust the significance level to perform multiple testing. **If statistically significant, continuous variables were analyzed by Kruskal-Wallis test followed by post-hoc analysis with Bonferroni adjustment to compare differences among PDD, DMD and PD-DMD groups and were prior adjusted for age using multivariate linear regression. Mann-Whitney U test were used to compare differences between PDD and PD-DMD groups. Categorical parameters were analyzed using χ2-test with Bonferroni adjustment for multiple testing.

Supplementary Table 3. Multivariable logistic regression analysis for risk factors of dementia in PD patients.

(a)

**Variables**	**Univariate**		**Multivariate model**	
	**OR (95%CI)**	**p**	**Adjusted OR (95% CI)**	**p**
**FBG<5.00 (mmol/L)**	**2.704(1.171,6.241)**	**0.020**	**4.380(1.748,10.975)**	**0.002**
**HCY>15.00 (μmol/L)**	**2.684(1.156,6.231)**	**0.022**	**3.131(1.243,7.888)**	**0.015**
**Hyperlipidemia**	2.448(0.980,6.119)	0.055	**3.075(1.142,8.277)**	**0.026**
**Age**	1.035(1.000,1.071)	0.050	**1.043(1.003,1.084)**	**0.034**
**Cys C>0.95 (mg/L)**	**2.122(1.008,4.468)**	**0.048**	2.157(0.979,4.752)	0.056

(b)

**Variables**	**Univariate**		**Multivariate model**	
	**OR (95%CI)**	**p**	**Adjusted OR (95% CI)**	**p**
**HCY>15.00 (μmol/L)**	**2.684(1.156,6.231)**	**0.022**	**2.522(1.042,6.104)**	**0.040**
**Hyperlipidemia**	2.448(0.980,6.119)	0.055	**2.638(1.018,6.840)**	**0.046**
Age	1.035(1.000,1.071)	0.050	1.030(0.992,1.069)	0.124
**Cys C>0.95 (mg/L)**	**2.122(1.008,4.468)**	**0.048**	2.057(0.947,4.464)	0.068
**Hypoglycaemic episodes (FBG≤3.9mmol/L)**	1.594(0.186,13.690)	0.671	2.949(0.308,28.262)	0.348

PD- patients with Parkinson disease without type 2 diabetes mellitus; OR- odds ratio; CI- confidence interval; FBG- fasting blood glucose; HCY- homocysteine; Cys C- Cystatin C.

Supplementary Table 4. Multivariable logistic regression analysis for risk factors of dementia in DM patients.

**Variables**	**Univariate**		**Multivariate model**	
	**OR (95% CI)**	**p**	**Adjusted OR (95% CI)**	**p**
**AST>40(IU/L)**	**6.074(1.913,19.286)**	**0.002**	**6.472(1.147,36.533)**	**0.034**
**Cys C>0.95 (mg/L)**	**3.864(1.584,9.424)**	**0.003**	**4.905(1.399,17.206)**	**0.013**
**Anxiety or depression**	**3.239(1.535,6.833)**	**0.002**	**3.750(1.220,11.526)**	**0.021**
**Male**	**2.549(1.125,5.775)**	**0.025**	**3.480(1.109,10.918)**	**0.033**
**Hypoglycaemic episodes (FBG≤3.9mmol/L)**	**5.062(1.208,21.212)**	**0.027**	7.013(0.903,54.463)	0.063
**Neutrophil (%)**	**1.054(1.017,1.093)**	**0.004**	1.151(0.983,1.348)	0.080
**Brain infarction**	**5.511(2.429,12.500)**	**<0.001**	2.485(0.788,7.834)	0.120
**Calcium<2.10(mmol/L)**	**5.414(1.892,15.490)**	**0.002**	3.464(0.694,17.294)	0.130
**Lymphocyte (%)**	**0.935(0.896,0.974)**	**0.001**	1.144(0.960,1.364)	0.133
**Potassium (mmol/L)**	**0.381(0.149,0.977)**	**0.045**	0.500(0.145,1.725)	0.273
**WMLs**	**4.450(2.166,9.142)**	**<0.001**	2.812(0.439,18.002)	0.275
**hs-CRP>3.00 (mg/L)**	**2.512(1.160,5.439)**	**0.019**	1.850(0.573,5.974)	0.303
**LDL-C<2.00(mmol/L)**	2.103(0.996,4.440)	0.051	1.712(0.615,4.770)	0.303
**SAE**	**5.421(2.618,11.224)**	**<0.001**	1.460(0.249,8.548)	0.675
**D-Dimer>0.50(mg/L)**	**3.698(1.522,8.986)**	**0.004**	1.309(0.318,5.383)	0.709
**Albumin<35.00(g/L)**	**5.339(1.513,18.846)**	**0.009**	1.207(0.207,7.026)	0.834
**Fibrinogen>4.00 (g/L)**	**2.431(1.066,5.543)**	**0.035**	1.098(0.306,3.947)	0.886
**Age**	**1.033(1.005,1.062)**	**0.022**	0.998(0.957,1.042)	0.931

DM- patients with type 2 diabetes mellitus; OR- odds ratio; CI- confidence interval; AST- Aspartate transaminase; Cystatin C- Cys C; FBG- Fasting blood glucose; WMLs- White matter lesions; hs-CRP- hypersensitive C-reactive protein; LDL-C- low density lipoprotein cholesterol; SAE- subcortical arteriosclerotic encephalopathy.

Supplementary Table 5. Multivariable logistic regression analysis for risk factors of dementia in PD (with and without DM) patients and the interaction of risk factors with the existence of DM.

Variables	Univariate		Multivariate Model		Interactioon
	OR (95%CI)	p	Adjusted OR (95% CI)	p	p
**Fibrinogen>4.00 (g/L)**	**3.002(1.677,5.374)**	**<0.001**	**3.259(1.674-6.345)**	**0.001**	0.979
**With DM**	**3.613(2.266,5.761)**	**<0.001**	**3.204(1.247,8.233)**	**0.016**	
**Without DM**	**0.277(0.174,0.441)**	**<0.001**	**3.267(1.059,10.082)**	**0.039**	
**LDL-C<2.00 (mmol/L)**	**2.129(1.142,3.969)**	**0.017**	1.544(0.783,3.043)	0.210	**0.022**
**With DM**	**2.586(1.594,4.197)**	**<0.001**	**3.347(1.305,8.585)**	**0.012**	
**Without DM**	**0.387(0.238,0.627)**	**<0.001**	0.384(0.078,1.890)	0.239	
**SAE**	**2.458(1.428,4.231)**	**0.001**	1.566(0.848,2.894)	0.152	**0.028**
**With DM**	**1.469(1.001,2.156)**	**0.049**	**3.952(1.566,9.971)**	**0.004**	
**Without DM**	**0.681(0.464,0.999)**	**0.049**	0.855(0.312,2.343)	0.761	
**FBG<5.00 (mmol/L)**	1.100(0.638,1.895)	0.732	**2.364(1.153,4.848)**	**0.019**	**0.025**
**With DM**	**0.108(0.067,0.175)**	**<0.001**	0.323(0.036,2.905)	0.313	
**Without DM**	**9.232(5.728,14.880)**	**<0.001**	**5.116(1.879,13.931)**	**0.001**	
**HCY>15.00 (μmol/L)**	**1.925(1.005,3.687)**	**0.048**	1.724(0.848,3.503)	0.132	0.163
**With DM**	0.907(0.554,1.1.486)	0.699	0.940(0.283,3.130)	0.920	
**Without DM**	1.102(0.673,1.804)	0.699	**2.828(1.068,7.492)**	**0.036**	
**Hyperlipidemia**	1.302(0.610,2.780)	0.495	1.391(0.618,3.133)	0.425	0.141
**With DM**	1.035(0.612,1.752)	0.898	0.717(0.146,3.506)	0.681	
**Without DM**	0.966(0.571,1.635)	0.898	**2.996(1.048,8.561)**	**0.041**	
**Diabetes**	1.685(0.991,2.863)	0.054	1.168(0.573,2.384)	0.669	
**Cys C>0.95 (mg/L)**	1.350(0.775,2.353)	0.290	1.267(0.681,2.355)	0.455	**0.022**
**With DM**	0.722(0.493,1.057)	0.094	0.467(0.155,1.405)	0.175	
**Without DM**	1.385(0.946,2.028)	0.094	**2.360(1.014,5.495)**	**0.046**	
**Age**	**1.051(1.024,1.078)**	**<0.001**	**1.044(1.010,1.078)**	**0.010**	0.854
**With DM**	**1.082(1.062,1.103)**	**<0.001**	1.059(0.999,1.123)	0.055	
**Without DM**	**0.924(0.907,0.942)**	**<0.001**	**1.052(1.007,1.099)**	**0.024**	

PD- patients with Parkinson disease without type 2 diabetes mellitus; DM- type 2 diabetes mellitus without Parkinson disease; OR- odds ratio; CI- confidence interval; LDL-C- low density lipoprotein cholesterol; SAE- subcortical arteriosclerotic encephalopathy; FBG- fasting blood glucose; HCY- homocysteine; Cys C- Cystatin C.
